# Numerical Analysis of Orbital Perturbation Effects on Inclined Geosynchronous SAR

**DOI:** 10.3390/s16091420

**Published:** 2016-09-02

**Authors:** Xichao Dong, Cheng Hu, Teng Long, Yuanhao Li

**Affiliations:** 1 School of Information and Electronics, Beijing Institute of Technology, Beijing 100081, China; cchchb@163.com (C.H.); longteng@bit.edu.cn (T.L.); lyh.900101@163.com (Y.L.); 2 Beijing Key Laboratory of Embedded Real-time Information Processing Technology, Beijing 100081, China

**Keywords:** geosynchronous SAR, orbital perturbation, focusing

## Abstract

The geosynchronous synthetic aperture radar (GEO SAR) is susceptible to orbit perturbations, leading to orbit drifts and variations. The influences behave very differently from those in low Earth orbit (LEO) SAR. In this paper, the impacts of perturbations on GEO SAR orbital elements are modelled based on the perturbed dynamic equations, and then, the focusing is analyzed theoretically and numerically by using the Systems Tool Kit (STK) software. The accurate GEO SAR slant range histories can be calculated according to the perturbed orbit positions in STK. The perturbed slant range errors are mainly the first and second derivatives, leading to image drifts and defocusing. Simulations of the point target imaging are performed to validate the aforementioned analysis. In the GEO SAR with an inclination of 53° and an argument of perigee of 90°, the Doppler parameters and the integration time are different and dependent on the geometry configurations. Thus, the influences are varying at different orbit positions: at the equator, the first-order phase errors should be mainly considered; at the perigee and apogee, the second-order phase errors should be mainly considered; at other positions, first-order and second-order exist simultaneously.

## 1. Introduction

Geosynchronous synthetic aperture radar (GEO SAR) [1] runs on an orbit height of around 36,000 km, has a revisit time of less than 24 h and a coverage of more than 1000 km by 1000 km. Recently, GEO SAR has become a hot topic, as it has overwhelming advantages for monitoring earthquakes and other disasters [2,3,4]. Related research is focused on the system design and optimization [5,6,7,8] and the development of accurate imaging algorithms [9,10]. Other studies are devoted to the non-ideal influences, including atmospheric effects [11,12,13,14]. However, orbital perturbations are rarely concerned, though satellites are certain to be impacted by perturbations caused by the Earth’s non-spherical mass distribution, the atmospheric drag, the third body attraction, the solar radiation, etc.

In spaceborne SAR, perturbations will lead to orbit drifts and variations by influencing the orbital elements (the parameters required to uniquely identify a specific orbit in celestial mechanics) varying with time. Perturbations imposed on satellites are related to orbit height. For the low Earth orbit (LEO) case, the *J*_2_ term perturbation (a kind of the Earth’s non-spherical perturbation) and the atmospheric drag should be mainly considered. Comparatively, the main sources of perturbations for the geosynchronous orbit are the *J*_2_ term perturbation and the third body attraction. The solar radiation should also be considered if the area/mass ratio is large. Furthermore, in LEO SAR, the radar image integration time is very short (at levels of 1 s), and thus, the orbit can be considered to be frozen within the integration time; while in GEO SAR, it behaves very differently. The perturbed orbit drifts among days or weeks can form the spatial baseline between the repeat pass GEO SAR and can be employed for the repeat pass interferometry or the differential interferometry [15,16]. In comparison, the perturbed orbit variations within one day or the GEO SAR aperture time (several hundreds or even thousands of seconds) can lead to the changing of the slant range histories and result in the degradation and even the failure of focusing [17,18].

Kou et al. [17] studies the perturbed orbit variations’ effects on the focusing and interferometry of the circular SAR (CSAR). However, the CSAR is an innovative SAR system, which has a circular nadir track and forms high resolution images by the coherent integration of the whole day’s (the orbit period) acquisitions. Therefore, its signal models and the corresponding conclusions cannot be used directly in the inclined GEO SAR, whose integration time is comparatively less and only at levels of around several minutes. In [18], the perturbation effects are analyzed for the GEO SAR in a circular geosynchronous orbit, and the approximate perturbed equations are adopted in analyzing the errors of the Doppler parameters and the focusing performance. The fourth-order Doppler parameters are adopted [19]. Therefore, for the more general cases of GEO SAR, which is running in the elliptical or near-circular orbit, the approximation method is not very accurate. In actuality, it is complex and difficult to derive the analytical solutions of the various perturbations’ influences on the orbital elements and the slant range histories because their effects are coupled with each other. In order to avoid the possible and great errors induced by approximation, numerical approaches are recommended, in which the AGI System Tool Kit (STK) (Analytical Graphics, Inc., Exton, PA, USA) software is employed to generate the highly accurate perturbations and the perturbed slant range histories.

This paper studies the perturbation influences on the orbital elements, the slant range histories and the focusing performance of GEO SAR based on the accurate numerical simulation using STK. However, the theory of the perturbation influences on the orbital elements is also demonstrated analytically. Then, the corresponding variations of slant range histories are derived based on the Taylor expansion of slant ranges. The analyses are based on the perturbed motion equations and the STK. The laws of the variations and the corresponding influences are deduced. As the analytical solution for perturbation dynamic equations and their composited influences on the slant range are difficult to obtain, a numerical approach based on STK is adopted to construct the SAR signals. The slant range histories with and without the effects of perturbations are generated. Finally, the focusing and evaluations are performed to validate the analyses based on the STK simulations.

## 2. Perturbations and Their Influences on GEO SAR Orbital Elements 

Satellites are influenced by the Earth’s non-spherical mass distribution, the atmospheric drag, the third body attraction, the solar radiation and others. The satellite orbit drifts induced by these perturbations can be depicted by their influences on the orbital elements based on the corresponding perturbation motion equations. In this section, all kinds of perturbations in GEO SAR are discussed analytically, along with the differences from those in LEO SAR. Then, the expressions, the periods and the orders of magnitude of the perturbations’ influences on orbit elements are presented. These will be used as a bridge to analyzing the perturbations’ influences on slant range histories in the next section.

### 2.1. Perturbation Motion Equations

**Figure 1:**
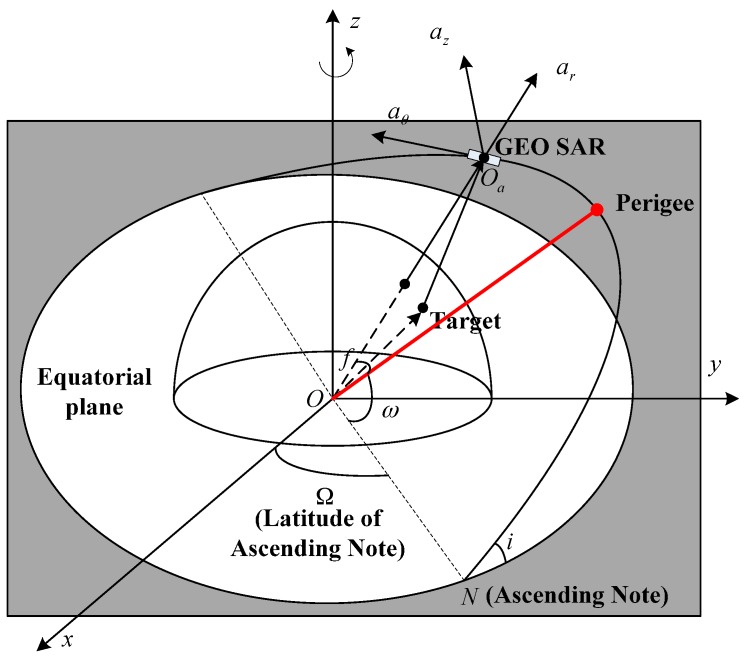
Geometry of the geosynchronous (GEO) SAR imaging. *O-xyz* is the Earth-centered inertial coordinate system. *O_a_-a_r_a_θ_a_z_* is the satellite local coordinate system.

For GEO SAR, perturbations will produce accelerations, which are composed of the radial $a_{r}$, the normal $a_{z}$ and the transverse $a_{\theta }$ components (following the axis order of $a_{\theta }$ $a_{z}$ and $a_{r}$, respectively). The radial component is along the direction pointing from the geocentric center to the satellite; the normal component is perpendicular to the orbit plane; and the transverse component lies within the orbit plane and follows the right-hand rule. The imaging geometry of GEO SAR is shown in Figure 1.

Equations of satellite motion have to be represented by perturbed equations. According to the orbital perturbation dynamic theory, the orbital elements influenced by the perturbations can be obtained. However, the Lagrangian perturbed equation can be only used for perturbation, which can be presented by a potential function (i.e., the Earth’s non-spherical perturbation and the third body perturbation). Considering the non-conservative perturbation forces, such as solar radiation and atmospheric drag, no potential functions exist for use; therefore, the Lagrangian perturbed equation of motion cannot be directly used in such a case. The Gaussian perturbed equation of motion can be employed for any type of perturbation. The temporal derivatives of the orbital elements have been expressed analytically [20]. The detailed algebras can be found in [20]. The differential equations are:

(1) $$\frac{da}{df}=\frac{p^{2}}{\mu }\frac{2p}{{\left(1-e^{2}\right)}^{2}}\left[\frac{e\;\sin\;f}{{\left(1+e\;\cos\;f\right)}^{2}}a_{r}+\frac{1}{1+e\;\cos\;f}a_{\theta }\right]$$

(2) $$\frac{de}{df}=\frac{p^{2}}{\mu }\left[\frac{\sin\;f}{{\left(1+e\;\cos\;f\right)}^{2}}a_{r}+\frac{e+2\cos\;f+e\;\cos^{2}\;f}{{\left(1+e\;\cos\;f\right)}^{3}}a_{\theta }\right]$$

(3) $$\frac{di}{df}=\frac{p^{2}}{\mu }\frac{\cos\left(f+\omega \right)}{{\left(1+e\;\cos\;f\right)}^{3}}a_{z}$$

(4) $$\frac{d\Omega }{df}=\frac{p^{2}}{\mu }\frac{1}{\sin\;i}\frac{\sin\left(f+\omega \right)}{{\left(1+e\;\cos\;f\right)}^{3}}a_{z}$$

(5) $$\frac{d\omega }{df}=\frac{p^{2}}{\mu }\left[-\frac{\cos\;f}{e{\left(1+e\;\cos\;f\right)}^{2}}a_{r}+\frac{\left(2+e\;\cos\;f\right)\sin\;f}{e{\left(1+e\;\cos\;f\right)}^{3}}a_{\theta }-\frac{1}{\tan\;i}\frac{\sin\left(f+\omega \right)}{{\left(1+e\;\cos\;f\right)}^{3}}a_{z}\right]$$

where p is the latus rectum and $p=a\left(1-e^{2}\right)$, μ is the geocentric gravitational constant. a, e, i, f, ω are the orbital elements and represent the semi-major axis, the eccentricity, the inclination, the true anomaly and the argument of perigee, respectively. The *d/df* is the differential operator.

### 2.2. Perturbations in GEO SAR

The various perturbations have different influences on satellites at different orbit heights [21]. For the low Earth orbit satellites, the *J*_2_ term accounts for about one percent of the two-body centripetal force. The atmospheric drag perturbation decreases as the satellite orbit height increases, and it is around the orders of $10^{-1}\sim 10^{-3}$ of the *J*_2_ term for most of the LEO satellites near 160–2,000 km orbit heights. When the orbit height becomes higher, its influences vanish. The third body attraction and the solar radiation perturbations are relatively small and are around the orders of 10^–4^~10^–5^ of the *J*_2_ term.

In comparison, the influences of perturbation on the high orbit satellites are very different. The *J*_2_ term will decrease as the orbit height increases; while the third body attraction increases (it will arrive at the same level as the *J*_2_ term at the geosynchronous orbit). The solar radiation perturbation remains almost unchanged. Furthermore, for any orbit height, the other perturbations, such as the tidal perturbation, are several orders smaller than the *J*_2_ term and, thus, can be ignored.

The influences of perturbation on GEO SAR are simulated using STK. The initial orbital elements of the reference GEO SAR have the semi-major axis of 42,164.2 km, the eccentricity of 0.07, the inclination of 53°, the right ascension of ascending node (RAAN) of 110° and the argument of perigee of 270°. The 10 days’ orbit drifts caused by each perturbation alone are produced and presented in Table 1. The orders of each perturbation’s influence are consistent with the aforementioned analysis. For GEO SAR, only the Earth’s non-spherical mass distribution, the third body attraction and the solar radiation should be considered.

Furthermore, the influences have obvious periodicity. According to the influences on GEO SAR orbital elements, perturbations can be categorized into the secular component and the periodical component. The secular component can cause the long-term and continuous drifts, while the periodical component will produce the periodical changes, including the short periodical term and the long periodical term. The short periodical term has a smaller period than the satellite orbit period, while the long periodical term has a much greater one. The periodical perturbations will cause oscillations around a certain position. For GEO SAR, the short period is within 24 h and is usually 12 h or 24 h. The long period can reach up to half a month, half a year or even several years.

### 2.3. Earth’s Non-Spherical Mass Distribution

In the ideal two-body movement (Keplerian motion), the satellite and the Earth are considered as two particles, and the gravity is calculated between them. However, the Earth is not a regular sphere, and its mass distribution is uneven, so the Earth’s gravity on satellites cannot be considered as the point-to-point form in real cases. If the Earth is considered as an irregular ellipsoid, which is composed of *n* particles approximately, the gravity on the satellite *P* outside the Earth is the sum of the gravities on *P* from *n* particles of the Earth. As the gravity is a vector, the summation is complex. Therefore, generally, the potential function *V* of the Earth’s gravitational field is firstly calculated, and then, the gravity is produced based on the potential function *V*.

According to the celestial mechanics, the series expansion of the potential function *V* is spherical harmonics series and can be presented as [22]:

(6) $$V\left(r,\varphi ,\lambda \right)=\frac{\mu }{r}\sum_{n=0}^{\infty }{\left(\frac{a_{E}}{r}\right)}^{n}\sum_{k=0}^{n}\left(C_{nk}\cos\;k\lambda +S_{nk}\sin\;k\lambda \right)P_{nk}\left(\sin\;\varphi \right)$$

where r,φ,λ is the geocentric range, the latitude and the longitude. $a_{E}$ is the Earth equatorial radius. $C_{nk}$ and $S_{nk}$ is the spherical harmonic coefficients. *n* and *k* are the orders. $P_{nk}\left(x\right)$ is the associated Legendre function, i.e.,:

(7) $$P_{nk}\left(x\right)={\left(1-x^{2}\right)}^{k/2}\frac{d^{k}}{dx^{k}}P_{n}\left(x\right)$$

while $P_{n}\left(x\right)$ is the Legendre polynomials:

(8) $$P_{n}\left(x\right)=\frac{1}{2^{n}n!}\frac{d^{n}}{dx^{n}}{\left(x^{2}-1\right)}^{n}$$

Measurements and calculations of these spherical harmonic coefficients are under research. Various models of the Earth gravitational field are constructed for the approximation to the Earth gravities. Currently, the latest model is the Earth Gravitational Model 2008 [23] (EGM2008, by the National Geospatial-Intelligence Agency, NGA), which has orders up to 2159 and a spatial resolution of 9 km. In the models, the lower order terms have a more important role in the models of the Earth gravitational field, especially for the second-order of zonal harmonics, i.e., the *J*_2_ term. Actually, the tesseral harmonics will induce the orbit precession and the longitude drifts. However, they are long periodical terms and will not influence focusing. When only the *J*_2_ term is considered, the Earth can be simplified as an ellipsoid with some oblateness. For the simplification for analyzing the mechanical model, only the *J*_2_ term is considered for influencing the orbital elements in this section. However, the other higher terms have similar impacts and analyzing methods.

When only the *J*_2_ term is considered, the perturbation term in the potential function *V* of the Earth’s gravitational field can be expressed as:

(9) $$V_{pert}\left(r,\varphi \right)=\frac{\mu {a_{E}}^{2}}{2r^{3}}J_{2}\left(3\sin^{2}\varphi -1\right)$$

where *J*_2_ is the Earth oblateness coefficient. The accelerations caused by perturbations can be expressed as:

(10) $$\vec{a}=-grad\cdot V_{pert}\left(r,\varphi \right)=-\left[\begin{array}{ccc} \vec{i_{r}} & \vec{i_{\theta }} & \vec{i_{z}} \end{array}\right]\left[\begin{array}{c} \frac{\partial }{\partial r}\\ \frac{\partial }{r\partial f}\\ \frac{\partial }{r\;\sin\left(f+\omega \right)\partial i} \end{array}\right]\cdot V_{pert}\left(r,\varphi \right)$$

where $\begin{array}{ccc} \vec{i_{r},} & \vec{i_{\theta },} & \vec{i_{z}} \end{array}$ are the unit vectors of the three orthogonal directions in the satellite local coordinate system.

Considering the sine law in the spherical triangle:

(11) sin φ=sin i sin(f+ω)

we can obtain the radial, the normal and the transverse components of the *J*_2_ perturbation accelerations:

(12) $$a_{r}=-\frac{3\mu {R_{e}}^{2}J_{2}}{2r^{4}}\left(1-3\sin^{2}i\;\sin2\left(f+\omega \right)\right)$$

(13) $$a_{\theta }=-\frac{3\mu {R_{e}}^{2}J_{2}}{2r^{4}}\sin^{2}i\;\sin2\left(f+\omega \right)$$

(14) $$a_{z}=-\frac{3\mu {R_{e}}^{2}J_{2}}{2r^{4}}\sin2i\;\sin\left(f+\omega \right)$$

Substituting Equations (12)–(14) into Equations (1)–(5), we can obtain the variation of the orbital elements under the influences of the *J*_2_ perturbation (see Appendix A for the detailed deviations):

(15) $$\frac{da}{df}=\frac{-3{R_{e}}^{2}J_{2}}{p{\left(1-e^{2}\right)}^{2}}{\left(1+e\;\cos\;f\right)}^{2}\left[e\;\sin\;f+\sin^{2}i\;\sin\;2u\left(1+e\;\cos\;f-3e\;\sin\;f\right)\right]$$

(16) $$\frac{di}{df}=\frac{-3{R_{e}}^{2}J_{2}\sin\;2i}{2p^{2}}\left(1+e\;\cos\;f\right)\sin\;u\;\cos\;u$$

(17) $$\frac{de}{df}=\frac{-3{R_{e}}^{2}J_{2}}{2p^{2}}{\left(1+e\;\cos\;f\right)}^{2}\left[\sin\;f+\sin^{2}\;i\sin\;2u\left(\cos\;f-3\sin\;f+\frac{e+\cos\;f}{1+e\;\cos\;f}\right)\right]$$

(18) $$\frac{d\Omega }{df}=\frac{-3{R_{e}}^{2}J_{2}\cos\;i}{p^{2}}\left(1+e\;\cos\;f\right)\sin^{2}u$$

(19) dωdf=−3Re2J22p2(1+e cos f)⋅dωdf[(1+e cos f)(sin f+cos f)+sin fesin2 i sin 2u−cos f(1+e cos f)e−2sin2 u cos2 i]

where u=f+ω.

Inferred from Equations (15)–(19), each orbital element has a short periodical term with a period of one GEO SAR orbit (T) under the influences of the *J*_2_ term. However, there exists no long periodical term. In comparison, the secular term will give a secular decreasing (↓) in the RAAN and a secular increasing (↑) in the argument of perigee. The specific law of the GEO SAR orbital elements variation is summarized in Table 2. The dash indicates no influence at all.

**Figure 2:**
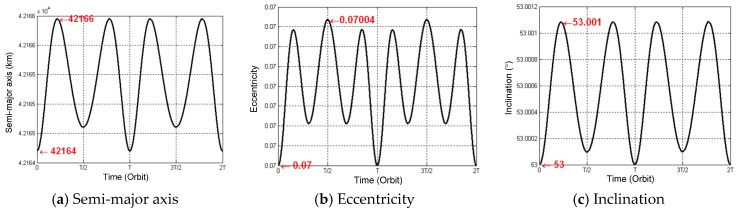

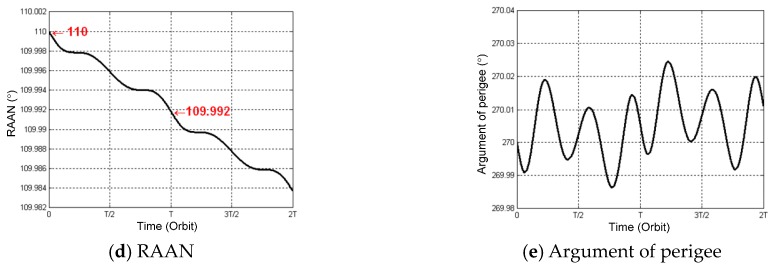
Curves of the short periodical terms of GEO SAR orbital elements’ variations under the influences of the *J*_2_ term.

The perturbation motion equations can be analytically solved by the canonical transformation, but the solving process is complicated and the accuracy limited. In this section, the numerical integral approach is adopted to solve the equations using MATLAB. The orbital elements variations are presented in Figure 2, which shows consistency with the aforementioned theoretical analysis. 

### 2.4. Attraction of the Sun and the Moon

The third body attraction perturbation is mainly caused by the attraction of the Sun and the Moon. Especially for GEO SAR, these perturbation effects should be considered. When the orbit height arises above 50,000 km, the influences of the attraction of the Sun and the Moon will exceed the *J*_2_ term.

The acceleration caused by the Moon attraction can be expressed as [22] (p. 65):

(20) $$\vec{a}=\frac{\mu_{M}\left(\vec{r_{M}}-\vec{r}\right)}{m{\left|\vec{r_{M}}-\vec{r}\right|}^{3}}-\frac{\mu_{M}\vec{r_{M}}}{m{r_{M}}^{3}}$$

where $\mu_{M}$ is the Moon's gravitational constant, $\vec{r_{M}}$ is the radial vector of the Moon in the Earth centered inertial coordinate system (ECI). $\vec{r}$ is the radial vector of the satellite in ECI. m is the satellite mass. The acceleration caused by the Sun attraction has a similar form as Equation (20).

The perturbation accelerations by the Sun and the Moon can also be decomposed into the radial, the normal and the transverse components and then be substituted into the perturbation motion Equations (1)–(5), obtaining the analytical variation forms. As the period of the Earth revolution around the Sun is one year and the period of the Moon revolution around the Earth is one month, the GEO SAR orbital elements variations under the influences of the third body perturbations (the Sun and the Moon attraction) have the long period term of half a month and half a year. If only the attraction of the Sun and the Moon is considered, the laws of the perturbed variations are summarized in Table 3.

### 2.5. Solar Radiation Pressure

Light illuminated on the object’s surface will produce pressure, which is named light pressure. Besides visible light, electromagnetic waves also have the light pressure interaction. The light pressure will impose perturbation forces on the satellite in space and will cause orbit drifts and variations. This kind of perturbation is called the light pressure perturbation in which the solar radiation perturbation contributes most. The accelerations caused by the solar radiation perturbation is expressed as:

(21) $$\vec{a}=-C_{r}\cdot \frac{A}{m}\cdot \frac{K\phi }{c}{\left(\frac{1}{r}\right)}^{2}\vec{\hat{r}}$$

where $C_{r}$ is the radiation pressure coefficient, which is generally set as between one and two. $C_{r}$ is defined as 1+ε, where ε is the reflectivity. When the incident power is perfectly absorbed, ε=0 and $C_{r}=1$; when the reflection is perfectly diffuse, $C_{r}=1.44$; when the reflection is perfectly specular, ε=1 and $C_{r}=2$. *A/m* is the satellite area-mass ratio; *K* is the illumination factor (i.e., one or zero depending on whether the satellite is in sunlight or not); ϕ is the light pressure acting at one astronomical unit from the Sun (constant); *c* is the light velocity; *r* is the distance between the satellite and the Sun; and $\vec{\hat{r}}$is the corresponding unit vector.

When ignoring the eccentricity of the Earth revolution and considering no shadow region (the sunlight incident angle is zero), the radial, the normal and the transverse components of the solar radiation perturbation accelerations can be expressed as:

(22) $$\vec{a_{r}}=-C_{r}\cdot \frac{A}{m}\cdot \frac{K\phi }{c}\left(\cos\;f_{⊙}\cos\;l+\sin\;f_{⊙}\cos\;i_{⊙}\sin\;i\right)$$

(23) $$\vec{a_{\theta }}=C_{r}\cdot \frac{A}{m}\cdot \frac{K\phi }{c}\left(\cos\;f_{⊙}\cos\;l-\sin\;f_{⊙}\cos\;i_{⊙}\sin\;i\right)$$

(24) $$\vec{a_{z}}=-C_{r}\cdot \frac{A}{m}\cdot \frac{K\phi }{c}\sin\;f_{⊙}\sin\;i_{⊙}$$

where l=θ+ω+Ω, $f_{⊙}$ is the true anomaly of the Sun in ECI and $i_{⊙}$ is the inclination of the Sun in ECI.

Substituting Equations (22)–(24) into the perturbation motion Equations (1)–(5), we can obtain the law of the GEO SAR orbital elements variations under the influences of the solar radiation perturbation. As the $f_{⊙}$ has a long period of one year, the orbital elements variations have an identical long period. The law of variations is listed in Table 4.

### 2.6. Influences on Orbital Elements

The compound influences of various perturbations on orbital elements and the corresponding periodical changing laws are summarized in Table 5. The short periodical variations are within one orbit period and can influence the GEO SAR slant range histories. The long periodical and secular variations will influence the coverage and locations.

The aforementioned analyses of the perturbations’ influences on GEO SAR are given based on the dynamic equations separately. However, in real cases, the perturbations impact GEO SAR simultaneously, and thus, the orbital changing rules under the total perturbations cannot be presented in analytical forms. Instead, the influences are calculated numerically. STK provides two numerical models, i.e., the high-precision orbit propagator (HPOP) and the long-term orbit predictor (LOP). The HPOP can accurately calculate the orbit under the comprehensive perturbation environment and is suitable for the short period and highly accurate orbit calculation. The LOP can calculate the averaged influences of perturbations and can reduce greatly the calculation time under some precision precondition.

LOP is suitable for the orbit calculation in much longer time scales, such as several months or several years. The changing rules of the GEO SAR orbit within five years are calculated using LOP in STK. Firstly, we will use STK to build a scene where a GEO SAR satellite is added. The orbit parameters of GEO SAR will be input as the attributes of the satellite. The initial orbit parameters of GEO SAR are as follows: the semi-major axis is 42,164.2 km; the eccentricity is 0.07; the orbit inclination is 53°; RAAN is 110°; and the argument of perigee is 270°. Then, the simulation parameters in Table are filled into STK for simulating perturbations. Here, several assumptions are made: (1) the eccentricity of the Earth’s revolution is not considered; (2) the shadow region of the Earth is not considered; (3) the sunlight is incident at the angle of 0°. In other words, the case of the maximum area-mass ratio is considered in the simulations; while in actual cases, the area-mass ratio could be variant and smaller. 

**Figure 3:**
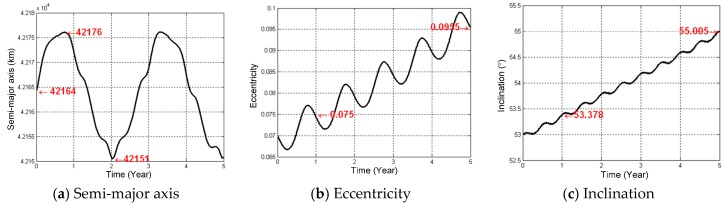

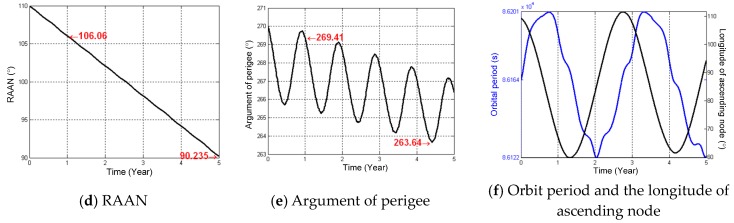
Curves of perturbed GEO SAR orbital elements variations within five years.

Simulation results are as shown in Figure 3. The orbital elements under the influences of the compound perturbations have the long and short periodical variations. All of the orbital elements, but the semi-major axis, have the secular variations. Under the influences of perturbations, the changing period of the semi-major axis is the same as that of the longitude drifts and the GEO SAR orbital period variations (around 2.7) years. In Figure 3f, the initial orbital period of GEO SAR is identical to the Earth rotation and is 86,164 s. When influenced by perturbations, the GEO SAR orbital period increases and becomes longer than the Earth rotation. Meanwhile, the longitude of the ascending node decreases and behaves as the nodal regression. After 1.38 years, the GEO SAR orbital period and the longitude of ascending node behave oppositely. Finally, this will lead to the reciprocating motion around a certain longitude and the variation range is around 50°, which is a function of the initial longitude relative to the stable longitude points, but obtained through the STK simulation here. The maximum value appears at the moment when the satellite is sent into orbit.

As the short periodical perturbations will influence focusing, the magnitudes of perturbed orbital elements are simulated based on the HPOP model in STK. The simulation parameters and options are similar to those in LOP, as shown in Table 6. The simulation start time is 4:00 a.m. on 1 May 2013 and the end time is 4:00 a.m. on 6 May 2013. After simulation, we will use a MATLAB connector to interface with STK. The perturbed orbit elements after a preset time interval can be output in MATLAB. Thus, the variation ranges of the orbital elements caused by the short periodical perturbations can be retrieved, and the results are listed in Table 7. 

## 3. Influences of Perturbations on GEO SAR Focusing

GEO SAR focusing is impacted by the slant range errors within the integration time. The slant range errors are caused by the variations of the perturbed GEO SAR orbital elements, consisting of the short periodical, the long periodical and the secular components. Because the long periodical and secular orbit drifts will impact the characteristics of the coverage and locations, they will not influence focusing and, thus, are not discussed in this section. In summary, this section carries out the modelling of slant ranges and then analyzes the slant range variations when each orbital element has a small variation of Δ, which is related to the short periodical perturbations.

### 3.1. GEO SAR Slant Range Model

In the Earth-centered Earth-fixed coordinate (ECEF) system, considering that the true anomaly at the aperture center moment *t*_0_ is *f*_0_, the coordinates of the point target being focused are:

(25) $${\vec{r}}_{t}\left(f_{0}\right)=\left(x_{t}\left(f_{0}\right),y_{t}\left(f_{0}\right),z_{t}\left(f_{0}\right)\right)$$

where:

(26) $$\left\{ \begin{array}{l} x_{t}=R_{e}\left[\cos\left(f+\omega \right)\cos\;\beta \cos\;\alpha -\sin\left(f+\omega \right)\sin\;\beta \cos\;i\cos\;\alpha -\sin\;\beta \sin\;i\sin\;\alpha \right]\\ y_{t}=R_{e}\left[\cos\left(f+\omega \right)\sin\;\beta \cos\;\alpha +\sin\left(f+\omega \right)\cos\;\beta \cos\;i\cos\;\alpha +\cos\;\beta \sin\;i\sin\;\alpha \right]\\ z_{t}=R_{e}\left[\sin\left(f+\omega \right)\sin\;i\cos\;\alpha -\cos\;i\sin\;\alpha \right] \end{array}\right.$$

where *R_e_* is the Earth radius, α is the geocentric angle and β equals $\Omega -\Omega_{G}$ and is related to the longitude. $\Omega_{G}$ is the Greenwich sidereal hour angle and equals $\Omega_{G0}+\omega_{e}t$, where $\Omega_{G0}$ is the initial Greenwich sidereal hour angle and $\omega_{e}$ is the Earth rotation angular velocity. *f* is the true anomaly; ω is the argument of latitude; *i* is the orbit inclination.

The coordinates of GEO SAR can be expressed as:

(27) $${\vec{r}}_{sf}=\left(x_{sf},y_{sf},z_{sf}\right)$$

where:

(28) $$\left\{ \begin{array}{l} x_{sf}=\cos\left(f+\omega \right)\cos\;\beta \cdot r-\sin\left(f+\omega \right)\sin\;\beta \cos\;i\cdot r\\ y_{sf}=\cos\left(f+\omega \right)\sin\;\beta \cdot r+\sin\left(f+\omega \right)\cos\;\beta \cos\;i\cdot r\\ z_{sf}=\sin\left(f+\omega \right)\sin\;i\cdot r \end{array}\right.$$

Therefore, the slant range can be calculated as:

(29) $$R=\sqrt{{\left[x_{sf}-x_{t}\left(f_{0}\right)\right]}^{2}+{\left[y_{sf}-y_{t}\left(f_{0}\right)\right]}^{2}+{\left[z_{sf}-z_{t}\left(f_{0}\right)\right]}^{2}}$$

The simplification of Equation (29) can be expressed as:

(30) $$R=\sqrt{r^{2}+{R_{e}}^{2}-2R_{e}r\cos\;\alpha_{0}}$$

where *R* is the slant range and *r* is the geocentric distance of GEO SAR and equals $a\left(1-e^{2}\right)/\left(1+e\;\cos\;f\right)$. $\alpha_{0}$ is the geocentric angle of the aperture center.

Inferred from Equation (30), *R* is mainly dependent on the semi-major axis and the eccentricity. When substituting the true anomaly *f* into Equation (30), *R* with respect to *f* can be considered as the range histories of GEO SAR. Assuming the range-Doppler algorithm is adopted, the location of the target will be influenced by *R*, as well. Therefore, the location will also be dependent on the semi-major axis and the eccentricity. Furthermore, the location will also be affected by the location of satellite and the beam pointing. If the location of the satellite changes while the beam pointing remains unchanged, location errors will be induced in the target location. Conclusively, the slant range history will be influenced by the semi-major axis and the eccentricity, while the location of target will be influenced by all of the orbit elements.

In order to analyze the influences of orbital elements’ variations on focusing, the GEO SAR slant ranges should be firstly Taylor expanded for retrieving Doppler parameters. Thus, the derivatives of the slant ranges will affect the focusing quality. The analytical forms of each order of the derivative of the slant range are obtained. Then, the influences of the orbital elements’ variations on focusing are analyzed. The slant range in Equation (30) is Taylor expanded to the third-orders at the aperture center, and we have:

(31) $$R\approx R_{0}+{\frac{dR}{dt}|}_{t=t_{0}}\left(t-t_{0}\right)+\frac{1}{2!}\cdot {\frac{d^{2}R}{dt^{2}}|}_{t=t_{0}}{\left(t-t_{0}\right)}^{2}+\frac{1}{3!}\cdot {\frac{d^{3}R}{dt^{3}}|}_{t=t_{0}}{\left(t-t_{0}\right)}^{3}$$

where *t*_0_ is the aperture center moment and *t* is the slow time.

The time derivatives of the slant range can be calculated as (refer to the Appendix for the detailed derivations):

(32) $$\frac{dR}{dt}=\dot{R}=\sqrt{\frac{\mu }{P}}\cdot \frac{e\;\sin\;f}{R}\left[r-R_{e}\;\cos\;\alpha_{0}\right]$$

(33) $$\frac{d^{2}R}{dt^{2}}=\ddot{R}=\frac{\mu }{PR}\left(e^{2}+e\;\cos\;f\right)-\frac{\mu }{r^{2}R}R_{e}\;\cos\;\alpha_{0}e\;\cos\;f-\frac{{\left(\dot{R}\right)}^{2}}{R}$$

(34) $$\begin{array}{c} \frac{d^{3}R}{dt^{3}}=\dddot{R}=-\sqrt{\frac{\mu^{3}}{P}}\cdot \frac{1}{r^{2}R}e\;\sin\;f-\frac{\mu \dot{R}}{PR^{2}}\cdot \left(e^{2}+e\;\cos\;f\right)+\frac{{\dot{R}}^{3}-2R\dot{R}\ddot{R}}{R^{2}}+\\ eR_{e}\;\cos\;\alpha_{0}\left(\sqrt{\frac{\mu^{3}}{P}}\frac{1}{r^{3}R}\left(\sin\;f+3e\;\sin\;f\;\cos\;f\right)+\frac{\mu \dot{R}}{r^{2}R^{2}}\cos\;f\right) \end{array}$$

At the aperture center where $t=t_{0}$ and $f=f_{0}$, we have:

(35) $${\frac{dR}{dt}|}_{t=t_{0}}=\sqrt{\frac{\mu }{P}}e\;\sin\;f_{0}\;\cos\;\theta_{L}\approx \mu^{1/2}\frac{e\;\sin\;f_{0}\;\cos\;\theta_{L}}{{r_{0}}^{1/2}}$$

(36) $$\begin{array}{ll} {\frac{d^{2}R}{dt^{2}}|}_{t=t_{0}} & =\mu \left(\frac{e^{2}-e^{2}\;\sin^{2}\;f_{0}\;\cos^{2}\;\theta_{L}+e\;\cos\;f_{0}}{r_{0}R_{0}\left(1+e\;\cos\;f_{0}\right)}-\frac{R_{e}\;\cos\;\alpha_{0}e\;\cos\;f_{0}}{{r_{0}}^{2}R_{0}}\right)\\ & \approx \mu \frac{e\;\cos\;f_{0}}{r_{0}R_{0}}\left(1-\frac{R_{e}\;\cos\;\alpha_{0}}{r_{0}}\right)\\ & =\mu \frac{e\;\cos\;f_{0}\;\cos\;\theta_{L}}{{r_{0}}^{2}} \end{array}$$

(37) $$\begin{array}{ll} {\frac{d^{3}R}{dt^{3}}|}_{t=t_{0}} & =\sqrt{\frac{\mu^{3}}{P}}\cdot \frac{e\;\sin\;f_{0}}{r_{0}R_{0}}\cdot \left[\begin{array}{l} -\frac{1}{r_{0}}+\frac{3e\;\cos\;\theta_{L}}{R_{0}}\left(\frac{e\;\sin^{2}\;f_{0}\;\cos^{2}\;\theta_{L}-e-\cos\;f_{0}}{1+e\;\cos\;f_{0}}\right)\\ +\frac{3e\;\cos\;\theta_{L}}{r_{0}R_{0}}\cos\;f_{0}R_{e}\;\cos\;\alpha_{0}+\frac{1}{{r_{0}}^{2}}\left(1+3e\;\cos\;f_{0}\right)R_{e}\;\cos\;\alpha_{0} \end{array}\right]\\ & \approx \sqrt{\frac{\mu^{3}}{P}}\cdot \frac{e\;\sin\;f_{0}}{r_{0}R_{0}}\cdot \left[\begin{array}{l} -\frac{1}{r_{0}}-\frac{3e\;\cos\;\theta_{L}\;\cos\;f_{0}}{R_{0}}\\ +\frac{3e\cos\;\theta_{L}\;\cos\;f_{0}}{R_{0}}\cdot \frac{R_{e}\;\cos\;\alpha_{0}}{r_{0}}+\frac{1}{{r_{0}}^{2}}\left(1+3e\;\cos\;f_{0}\right)R_{e}\;\cos\;\alpha_{0} \end{array}\right]\\ & =-\sqrt{\frac{\mu^{3}}{P}}\cdot \frac{e\;\sin\;f_{0}\;\cos\;\theta_{L}\left(1+3e\;\cos\;f_{0}\right)}{{r_{0}}^{2}}\\ & \approx -\mu^{3/2}\frac{e\;\sin\;f_{0}\;\cos\;\theta_{L}}{{r_{0}}^{7/2}} \end{array}$$

In calculating Equations (35)–(37), $R_{e}\;\cos\;\alpha_{0}$ is the projection of Earth radius *R_e_* to the geocentric range of GEO SAR. Therefore, we have the relationship of $\cos\;\theta_{L}={\left(r_{0}-R_{e}\;\cos\;\alpha_{0}\right)/R}_{0}$ where $\theta_{L}$ is the down-look angle.

Substituting Equations (35)–(37) into Equation (31), we have:

(38) $$\begin{array}{ll} R & \approx R_{0}+\mu^{1/2}\frac{e\;\sin\;f_{0}\;\cos\theta_{L}}{{r_{0}}^{1/2}}\left(t-t_{0}\right)+\mu \frac{e\;\cos\;f_{0}\;\cos\theta_{L}}{2{r_{0}}^{2}}{\left(t-t_{0}\right)}^{2}\\ & -\mu^{3/2}\frac{e\;\sin\;f_{0}\cos\theta_{L}}{6{r_{0}}^{7/2}}{\left(t-t_{0}\right)}^{3}\\ & =R_{0}+C_{1}\left(t-t_{0}\right)+C_{2}{\left(t-t_{0}\right)}^{2}+C_{3}{\left(t-t_{0}\right)}^{3} \end{array}$$

where $C_{i}$ can be expressed as:

(39) $$\begin{array}{l} C_{1}=\mu^{1/2}\frac{e\;\sin\;f_{0}\;\cos\theta_{L}}{{r_{0}}^{1/2}}\\ C_{2}=\mu \frac{e\;\cos\;f_{0}\;\cos\theta_{L}}{2{r_{0}}^{2}}\\ C_{3}=-\mu^{3/2}\frac{e\;\sin\;f_{0}\;\cos\theta_{L}}{6{r_{0}}^{7/2}}. \end{array}$$

In order to validate the slant range model, we will check the phase errors induced by the Taylor expansion to approximate the ideal slant range as the above discussion. The simulation parameters of the imaging validation are listed in Table 8. It should be noted that for the highly inclined GEO SAR (with inclination of 53°), the integration time of around 100 s can achieve a moderate resolution of 20 m.

**Figure 4:**
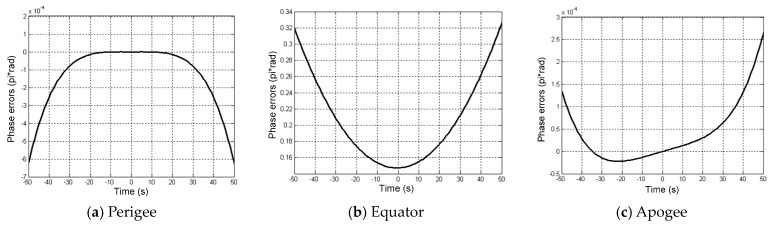
Phase errors of the Taylor expansion approximation to the ideal GEO SAR slant range.

Assuming the GEO SAR transmitting and receiving slant ranges are identical to *R*, the phase errors when using the Taylor expansion are shown in Figure 4. Inferred from Figure 4, the phase errors are at the orders of $10^{-4}\pi$ at perigee and apogee. The maximum phase error at the equator is 0.32π. Thus, the phase errors are all below the theoretical threshold of π/4. The approximation can satisfy the analysis of the influences of the orbital elements variations on the derivatives of the slant range.

### 3.2. Influences of Orbital Elements on Slant Range

As the aforementioned analysis, only the semi-major axis and the eccentricity should be considered in analyzing influences on the GEO SAR slant ranges. Therefore, in this section, only the errors of the slant range derivatives *C*_1_, *C*_2_ and *C*_3_ are derived when the variations of the semi-major axis and the eccentricity exist.

#### 3.2.1. Influences of the Semi-Major Axis

When the semi-major axis has a small variation of Δa, all orders of the slant range coefficient can be approximated by the first derivative:

(40) $$\Delta C_{ia}\approx \frac{dC_{i}}{da}\Delta a$$

where $C_{i}$ can be referred to Equation (38) and $r_{0}=a\left(1-e^{2}\right)/\left(1+e\;\cos\;f_{0}\right)$. It can be transformed into (see Appendix A for the detailed deviations):

(41) $$\Delta C_{1a}\approx \frac{dC_{1}}{da}\Delta a=-\frac{1}{2}\cdot \frac{C_{1}}{a}\cdot \Delta a$$

(42) $$\Delta C_{2a}\approx \frac{dC_{2}}{da}\Delta a=-\frac{C_{2}}{a}\cdot \Delta a$$

(43) $$\Delta C_{3a}\approx \frac{dC_{3}}{da}\Delta a=\frac{7}{12}\cdot \frac{C_{3}}{a}\cdot \Delta a$$

**Figure 5:**
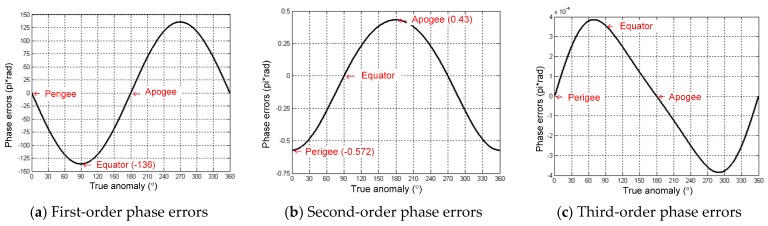
Phase errors of one orbit along with the true anomaly caused by the variations of semi-major axis Δ*a*.

According to Figure 3a, Δa has a long periodical variation with a maximum range of 25 km. Therefore, all orders of the slant range coefficient can be produced as the maximum value of Δa = 25 km, and the synthetic aperture time is 100 s. The corresponding results are shown in Figure 5. The phase errors are almost the same for all of the down-look angles. Therefore, the results in Figure 5 take the case of the down-look angle of 4.65°. From Figure 5, the third-order phase errors are below $4\times 10^{-4}\pi$ and can be ignored. At the perigee and apogee, Δa mainly leads to the second-order phase errors. At the equator, Δa mainly leads to the first-order phase errors. At other orbit positions, Δa can produce both the first-order and the second-order phase errors.

#### 3.2.2. Influences of Eccentricity

When the eccentricity has a small variation of Δe, all orders of the slant range coefficient can be approximated by (see Appendix A for the detailed deviations):

(44) $$\Delta C_{1e}\approx \frac{dC_{1}}{de}\Delta e=C_{1}\left(\frac{1}{e}-\frac{1}{2r}\cdot \frac{dr}{de}\right)\Delta e$$

(45) $$\Delta C_{2e}\approx \frac{dC_{2}}{de}\Delta e=C_{2}\left(\frac{1}{e}-\frac{2}{r}\cdot \frac{dr}{de}\right)\Delta e$$

(46) $$\Delta C_{3e}\approx \frac{dC_{3}}{de}\Delta e=C_{3}\left(\frac{1}{e}-\frac{7}{2r}\cdot \frac{dr}{de}\right)\Delta e$$

**Figure 6:**
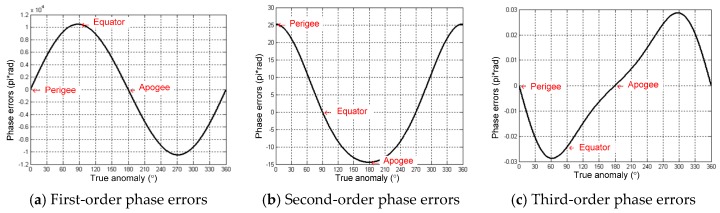
Phase errors of one orbit along with the true anomaly caused by the variations of eccentricity Δ*e*.

The variations of all orders of the slant range coefficient caused by Δe are at the levels of $10^{3}\Delta e$, $10^{-1}\Delta e$ and $10^{-6}\Delta e$. According to Figure 3b, Δe has a secular variation, and the annual deviation is around 0.005. Therefore, in the cases of Δe=0.005 and the synthetic aperture time of 100 s, all orders’ phase errors caused by Δe are calculated and presented in Figure 6. The phase errors are also almost the same for all of the down-look angles. 

Therefore, the results in Figure 6 take the case of the down-look angle of 4.65°. From Figure 6, the third-order phase errors are below 0.03π and can be ignored. At the perigee and apogee, Δe mainly leads to the second-order phase errors. At the equator, Δe mainly leads to the first-order phase errors. At other orbit positions, Δe can produce both the first-order and the second-order phase errors.

Conclusively, the semi-major axis and the eccentricity variations caused by perturbations can result in the errors of the first-order and second-order of the slant range and, thus, the corresponding first-order and second-order phase errors, leading to the focusing degradation. The third-order phase errors or above will not affect the focusing quality.

### 3.3. Influences of Orbital Elements Variations on Focusing

According to the focusing theory, all orders of phase errors except the constant phase could affect the focusing. The first-order will only lead to image drifts, while the second-order will change the frequency modulation rate *f_dr_* and leads to the focusing degradation, including the broadening and declination of the main lobe, along with the sidelobes arising. The third-order will result in the asymmetrical sidelobes and the broadening main lobe.

The point targets at perigee and equator are selected for validating the influences of orbital elements variations on focusing. The focusing algorithms employing the series reversion [24] will be used. In the simulation, the initial ideal two body motion and the slant ranges are firstly obtained using STK. Then the perturbed slant ranges after an increment of Δa for the semi-major axis and Δe for the eccentricity are also produced using STK. The ideal two body slant ranges will be used as reference to match the perturbed slant ranges and focus. The simulation parameters of the imaging validation are referred to Table 8. According to the simulation in Table 7, the variations of the semi-major axis and the eccentricity caused by perturbations will be 5288 m and 1.7 × 10^−4^. So in the imaging validation, Δa and Δe are set as 5300 m and 1.7 × 10^−4^, respectively.

**Figure 7:**
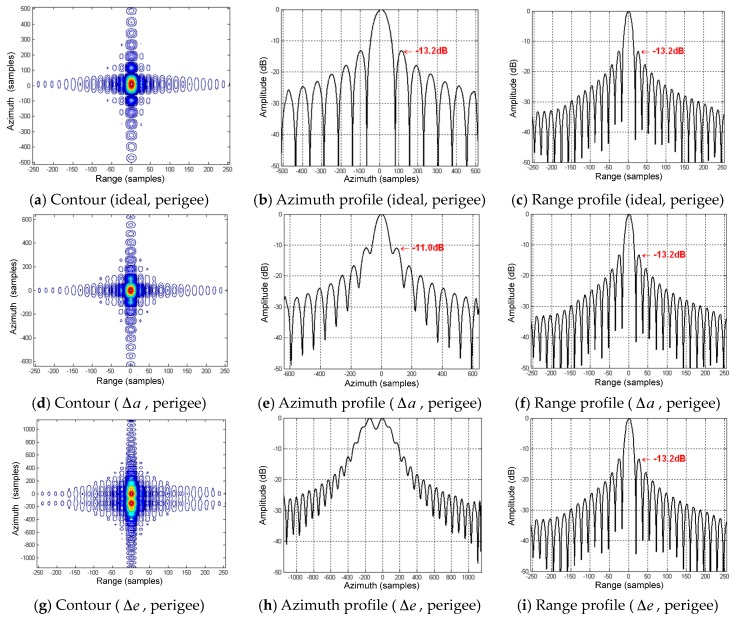

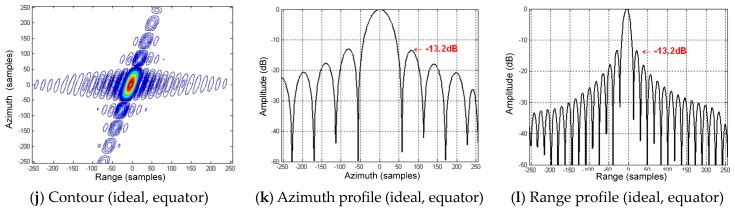
Perturbed imaging results of the simulated point targets in GEO SAR when considering the variations of the semi-major axis and eccentricity.

As shown in Figure 7a–c, it can be well focused in the range and azimuth at perigee if no variations are added in the orbital elements, and the peak sidelobe ratios (PSLR) can achieve the ideal level of −13.2 dB. As shown in Figure 7d–f, when an increment of 5300 m is added to the semi-major axis (i.e., it rises from 42,164.2–42,169.5 km), it cannot be well focused in the azimuth at perigee. The sidelobes rises seriously, and the azimuth PSLR is deteriorated to −11.0 dB. The focusing in range is not affected. Meantime, the target remains at the scene center, and no asymmetrical sidelobes exist. This suggests that Δa only results in the second-order of the slant range without the first-order and second-order variations. As shown in Figure 7g–i, when an increment of 0.00017 is added to the eccentricity (i.e., it rises from 0.07–0.07017), it cannot be focused at all in the azimuth at perigee. The main lobe has been overwhelmed by the sidelobes, but the target is still in the scene center, and no asymmetrical sidelobes exist either. The range focusing is good. This suggests a similar variation as that for Δa, and only the second-order slant range is changed.

For the focusing at the equator, the results are shown in Figure 7j–l. It can be well focused in the range and azimuth when no orbital elements’ variations exist. When the variations Δa and Δe are considered, only the location of the point target is changed without any influence on focusing. Thus, it can be concluded that only Δa and Δe change the first-order slant range of GEO SAR at the equator. The focusing quality is not impacted, and thus, the perturbed imaging results are not shown here.

In conclusion, the GEO SAR slant range is mainly related to the semi-major axis and eccentricity. The variations of these two items will result in the errors of the first-order and second-order slant ranges, while the influences of the third-order and above could be ignored. At different orbit positions, the influences have different behaviors. At the equator, the first-order phase errors should be mainly considered; at the perigee and apogee, the second-order phase errors should be mainly considered; at other positions, the first-order and second-order phase errors exist simultaneously.

## 4. STK Simulation and Verification

The perturbed GEO SAR slant range history and the accurate signal model cannot be obtained analytically from the GEO SAR geometry and the perturbation dynamic equations directly. For the second best, the perturbed signals can be generated by using the deduced variations of the slant ranges. This is useful in summarizing the changing laws based on the error propagation theory, but not very convincing in analyzing the influences of perturbations on GEO SAR accurately. Instead, the numerical approach is a good alternative in simulating the influence directly. In this section, the HPOP in STK is used to simulate the perturbed GEO SAR slant range histories and then to generate the echoes. The series reversion algorithm is adopted for focusing. The simulation parameters are the same as in Table 8. 

**Figure 8:**
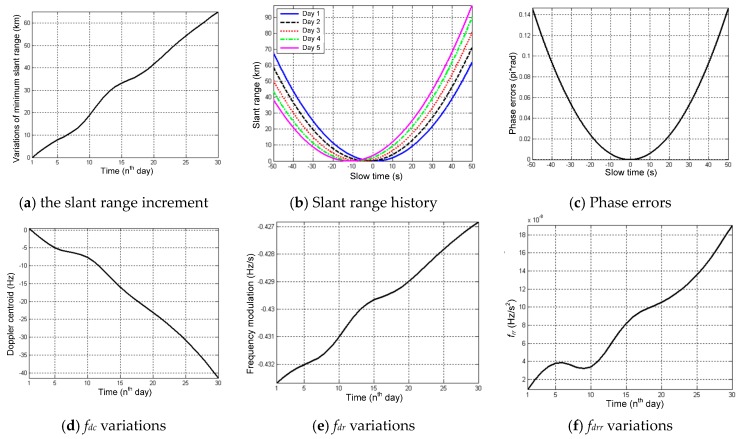
Variations of the slant range history and the Doppler coefficient (Doppler centroid: *f_dc_*, frequency modulation rate: *f_dr_* and third-order Doppler rate: *f_drr_*) of the perturbed GEO SAR within one aperture at perigee.

The simulation results are presented in Figure 8. Figure 8a gives the drifts of the minimum slant range (equivalent to the range at the aperture center). The derivations from the initial position increase continually within 30 days and accumulate up to 65 km at perigee (in comparison with 34 km at the equator). Ignoring the constant component in the slant range, the perturbed slant range histories within the 100-s aperture time are presented in Figure 8b. The variations are changing for 1–5 days from the initial position. The phase errors caused by perturbations within the integration time of 100 s can achieve up to 0.14π, as shown in Figure 8c. If the aperture increases, the phase errors also deteriorate and even have to be considered when achieved up to a certain extent.

Figure 8d–f shows the variations of the first-order to the third-order Doppler coefficient, which will influence focusing. The first-order Doppler rate coefficient is the Doppler centroid *f_dc_*; the second-order is the Doppler modulation rate *f_dr_*; and the third-order is the second derivatives of the Doppler history *f_drr_*. At perigee, the maximum increment of *f_drr_* after 30 days is up to 2 × 10^–7^ Hz/s^2^ within the integration time of 100 s. Correspondingly, the accumulated phase error within 100 s is only 8 × 10^–3^π, which is far less than the threshold of π/4 in SAR theory. Thus, the influence of *f_drr_* can be ignored. In comparison, the variations of *f_dc_* are non-zero and will induce image drifts; the variations of *f_dr_* would result in accumulated quadratic errors of above π/4, which will cause defocusing. Conclusively, the variations of *f_dc_* and *f_dr_* should be considered.

**Figure 9:**
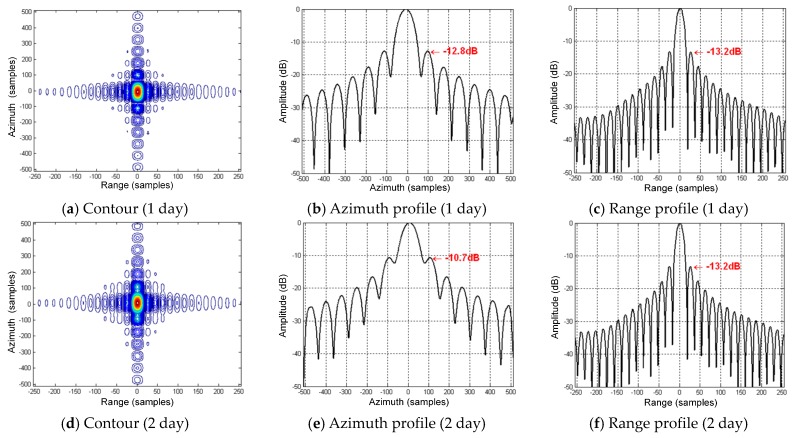

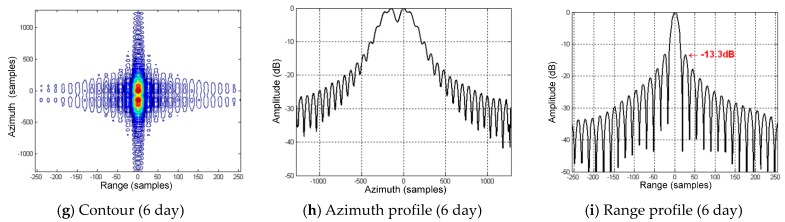
Imaging results of the perturbed GEO SAR point targets at perigee after the 1, 2 and 6 days’ perturbations.

Figure 9 shows the perturbed imaging results of point targets at perigee after the 1, 2 and 6 days’ perturbations. The perturbation can produce the phase errors within one aperture and is certain to degrade the focusing. The azimuth PSLR of the imaging result after one day (see Figure 9a–c) will drop to −12.8 dB, while the range focusing is good. As for the imaging results after two days in Figure 9d–f, the sidelobes rise apparently; the azimuth PSLR drops to −10.7 dB; and the point target deviates from the scene center. This means that there exist obvious first-order and second-order phase errors. As for the imaging results after six days in Figure 9g–i, there is serious defocusing in the azimuth, and the drift is also serious. In comparison, the focusing in range is not impacted. The evaluations of the imaging results of the perturbed GEO SAR point targets at perigee are listed in Table 9.

## 5. Conclusions

Perturbation is a main error source influencing GEO SAR focusing. It will cause the variations of GEO SAR orbit elements, among which the changes of semi-major axis and eccentricity can result in the varying slant range histories. The studies about perturbation influences on the orbital elements and the slant range histories are carried out analytically based on the perturbed motion equations and the Taylor expansion approximation. The changing laws of the perturbed orbital elements and the slant range variations are deduced, along with the corresponding influences. The variations of the perturbed GEO SAR slant range will induce the first-order and the second-order errors within the integration time. Thus, the accumulated linear and quadratic phase errors will deteriorate the focusing quality.

The focusing performance is analyzed based on the numerical approach using STK, which is adopted to construct the SAR signals with and without the effects of perturbations. Then, the imaging results are evaluated and compared. The simulations have good consistency with the aforementioned theoretical analyses that the first-order and second-order phase errors should be considered, while the cubic and the higher-order phase errors will not impact the imaging and, thus, can be ignored. For the GEO SAR (with the elliptical orbit, the inclination of 53° and the argument of perigee of 90°), the influences are dependent on the geometry configurations (as the Doppler parameters and the integration time are different). Therefore, at different orbit positions, the influences have different behaviors. At the equator, the first-order phase errors should be mainly considered; at perigee and apogee, the second-order phase errors should be mainly considered; at other positions, the first-order and second-order exist simultaneously. Though these conclusions are derived from the specific reference GEO SAR orbit chosen, the numerical approach can be generalized to the GEO SAR applications. In operation, the perturbations’ influences will accumulate during the mission life, and thus, the performance will deteriorate. When the perturbation errors become intolerable, it is recommended to compensate them through accurate measurements.

Actually, orbit maintenance is also a possible compensation alternative. It is a direct way to impose a certain force, which counteracts the perturbation forces. Correspondingly, the perturbed motion equations can be modified by adding such a force. However, in space missions, one limiting factor is fuel consumption for orbit maintenance. If the fuel runs out, the space missions will be degraded or even fail. Thus, though continuous orbit maintenance would be necessary for compensating the influences of orbit oscillations on focusing, the demands for fuel will be huge and beyond tolerance. Resultantly, continuous maintenance is not the option. Furthermore, GEO SAR has a huge platform and a large antenna, which are employed for compensating the huge slant range loss. Therefore, if continuous orbit maintenance is employed, it is difficult to stabilize the platform within the focusing time. The focusing performance can be degraded seriously in this case. 

In summary, orbit maintenance is only preferred when the accumulated orbit drifts caused by the long-term periodical and secular perturbations are achieved up to a certain level when the observation plan is impacted. If so, the operation life time can be prolonged. For improving GEO SAR focusing influenced by perturbations, it is recommended to employ accurate orbit measurements or some signal processing methods, such as the phase gradient autofocus (PGA) algorithm.

## Figures and Tables

**Table 1:** GEO SAR orbit drifts after 10 days when considering each perturbation alone.

Earth’s Non-Spherical Mass Distribution		Third Body Attraction	Solar Radiation	Others (Tidal Perturbation)	Total
*J*_2_ Term	Orders of 70 × 70				
105.3 km	141.3 km	95.9 km	34.2 km	0.008 km	227.8 km

**Table 2:** Law of the GEO SAR orbital elements variations under the influences of the *J*_2_ term

Orbital Elements	Short Period	Long Period	Secular
Semi-major axis	T	-	-
Eccentricity	T	-	-
Inclination	T	-	-
RAAN	T	-	↓
Argument of perigee	T	-	↑

Note: T is the period of GEO SAR; ↑ is increase; ↓ is decrease.

**Table 3:** Law of the GEO SAR orbital elements’ variations under the influences of the third body perturbations.

Orbital Elements	Short Period	Long Period	Secular
Semi-major axis	T	0.5 months/0.5 years	-
Eccentricity	T	0.5 months/0.5 years	↑
Inclination	T	0.5 months/0.5 years	↑
RAAN	T	0.5 months/0.5 years	↓
Argument of perigee	T	0.5 months/0.5 years	↓

**Table 4:** Law of GEO SAR orbital elements’ variations under the influences of the solar radiation perturbation.

Orbital Elements	Short Period	Long Period	Secular
Semi-major axis	T	1 year	-
Eccentricity	T	1 year	-
Inclination	T	1 year	↑
RAAN	T	1 year	↓
Argument of perigee	T	1 year	-

**Table 5:** Summary of the periodical changing laws of the perturbed GEO SAR orbital elements variations (SP: short periodical term, LP: long periodical term, S: secular term).

Orbital Elements	Earth’s Non-Spherical Mass Distribution	Attraction of Moon and Sun	Solar Radiation Pressure
Semi-major axis	SP	SP + LP	SP + LP
Eccentricity	SP	SP + LP + S↑	SP + LP
Inclination	SP	SP + LP + S↑	SP + LP + S↑
RAAN	SP + S↓	SP + LP + S↓	SP + LP + S↓
Argument of perigee	SP + S↑	SP + LP + S↓	SP + LP

**Table 6:** STK parameters and options for the perturbations’ simulation.

Parameters	Values	Parameters	Values
Simulation start time	0:00 a.m. 1 July 2007	Simulation end time	12:00 p.m. 11 July 2012
Coordinate system	J2000	Earth gravity model	EGM2008(70 × 70)
Solar radiation model	Spherical	Surface reflectivity	1.3
Area-mass ratio	0.31416 m^2^/kg	The third body attraction	The Sun/Moon

**Table 7:** Ranges of the orbital elements variations caused by the simulated short periodical perturbations.

Orbital Elements	Earth’s Non-Spherical Mass Distribution	Attraction of Moon and Sun	Solar Radiation Pressure	Others	Total
Semi-major axis (m)	2308	2900	1255	0.093	5288
Eccentricity	3.7 × 10^−5^	1.2 × 10^−4^	6.8 × 10^−5^	1.2 × 10^−9^	1.7 × 10^−4^
Inclination (°)	1.1 × 10^−3^	2.9 × 10^−3^	3.4 × 10^−4^	7.6 × 10^−8^	3.4 × 10^−3^
RAAN (°)	8.1 × 10^−3^	1.5 × 10^−3^	4.9 × 10^−4^	3.7 × 10^−8^	7.1 × 10^−3^
Argument of perigee (°)	0.032	0.082	0.013	1.5 × 10^−6^	0.111

**Table 8:** Simulation parameters of the imaging validation experiment.

Parameters	Values	Unit	Parameters	Values	Unit
Semi-major axis	42,164.2	km	Down-look angle	4.65	°
Eccentricity	0.07	-	Antenna diameter	24	m
Inclination	53	°	Wavelength	0.24	m
RAAN	105	°	PRF	200	Hz
Argument of perigee	270	°	Integration time	100	s
Mean anomaly	0	°	Pulse width	20	us
Δ*a*	5300	m	Signal bandwidth	18	MHz
Δ*e*	0.00017	-	Sampling rate	20	MHz

**Table 9:** Evaluations of the perturbed GEO SAR point targets at perigee.

	PSLR (dB)		ISLR (dB)		Drift (m)
	Range	Azimuth	Range	Azimuth	Azimuth
Ideal two body motion	−13.20	−13.22	−9.94	−10.36	0
1 day’s perturbation	−13.18	−10.94	−9.93	−8.02	2
2 days’ perturbation	−13.18	−10.7	−9.92	−6.91	1413
6 days’ perturbation	−13.20	−13.19	−10.11	−10.87	5727

## Appendix A

The differential equations of the orbital elements variations under the influences of the *J*_2_ perturbation can be obtained by substituting Equations (12)–(14) into Equations (1)–(5). Considering the relationship between the latus rectum *p* and the radium *r*, which is:

(A1) $$r=\frac{p}{1+e\;\cos\;f},$$

Equations (15)–(19) can be derived by the following algebra:

(A2) $$\begin{array}{ll} \frac{da}{df} & =-\frac{p^{2}}{\mu }\frac{2p}{{\left(1-e^{2}\right)}^{2}}\frac{3\mu {R_{e}}^{2}J_{2}}{2r^{4}}\left[\begin{array}{l} \frac{e\;\sin\;f}{{\left(1+e\;\cos\;f\right)}^{2}}\left(1-3\sin^{2}i\;\sin\;2\left(f+\omega \right)\right)\\ +\frac{1}{1+e\;\cos\;f}\sin^{2}i\;\sin\;2\left(f+\omega \right) \end{array}\right]\\ & =-\frac{3{R_{e}}^{2}J_{2}}{p{\left(1-e^{2}\right)}^{2}}\frac{p^{4}}{r^{4}}\frac{1}{{\left(1+e\;\cos\;f\right)}^{2}}\left[\begin{array}{l} e\;\sin f\left(1-3\sin^{2}i\;\sin\;2u\right)\\ +\left(1+e\;\cos\;f\right)\sin^{2}i\;\sin\;2u \end{array}\right]\\ & =-\frac{3{R_{e}}^{2}J_{2}}{p{\left(1-e^{2}\right)}^{2}}{\left(1+e\;\cos\;f\right)}^{2}\left[\begin{array}{l} e\;\sin\;f-3e\;\sin\;f\sin^{2}i\;\sin\;2u\\ +\sin^{2}i\;\sin\;2u+e\;\cos\;f\sin^{2}i\;\sin\;2u \end{array}\right]\\ & =-\frac{3{R_{e}}^{2}J_{2}}{p{\left(1-e^{2}\right)}^{2}}{\left(1+e\;\cos\;f\right)}^{2}\left[e\;\sin\;f+\sin^{2}i\;\sin\;2u\left(1+e\;\cos\;f-3e\;\sin\;f\right)\right] \end{array}$$

(A3) $$\begin{array}{ll} \frac{de}{df} & =-\frac{p^{2}}{\mu }\left[\begin{array}{l} \frac{\sin\;f}{{\left(1+e\;\cos\;f\right)}^{2}}\frac{3\mu {R_{e}}^{2}J_{2}}{2r^{4}}\left(1-3\sin^{2}i\;\sin\;2\left(f+\omega \right)\right)\\ +\frac{e+2\cos\;f+e\;\cos^{2}\;f}{{\left(1+e\;\cos\;f\right)}^{3}}\frac{3\mu {R_{e}}^{2}J_{2}}{2r^{4}}\sin^{2}i\;\sin\;2\left(f+\omega \right) \end{array}\right]\\ & =-\frac{p^{2}}{\mu }\frac{3\mu {R_{e}}^{2}J_{2}}{2r^{4}}\left[\begin{array}{l} \frac{\sin\;f}{{\left(1+e\;\cos\;f\right)}^{2}}\left(1-3\sin^{2}i\;\sin\;2\left(f+\omega \right)\right)\\ +\frac{e+2\cos\;f+e\;\cos^{2}\;f}{{\left(1+e\;\cos\;f\right)}^{3}}\sin^{2}i\;\sin\;2\left(f+\omega \right) \end{array}\right]\\ & =-\frac{3{R_{e}}^{2}J_{2}}{2p^{2}}\frac{p^{4}}{r^{4}}\frac{1}{{\left(1+e\;\cos\;f\right)}^{3}}\left[\begin{array}{l} \sin f\left(1+e\;\cos\;f\right)\left(1-3\sin^{2}i\;\sin\;2\left(f+\omega \right)\right)\\ +\left(e+2\cos\;f+e\;\cos^{2}\;f\right)\sin^{2}i\;\sin\;2\left(f+\omega \right) \end{array}\right]\\ & =-\frac{3{R_{e}}^{2}J_{2}}{2p^{2}}\left(1+e\;\cos\;f\right)\left[\begin{array}{l} \sin\;f\left(1-3\sin^{2}i\;\sin\;2u+e\;\cos\;f-3e\;\cos\;f\sin^{2}i\;\sin\;2u\right)\\ +\left(e+2\cos\;f+e\;\cos^{2}\;f\right)\sin^{2}i\;\sin\;2u \end{array}\right]\\ & =-\frac{3{R_{e}}^{2}J_{2}}{2p^{2}}\left(1+e\;\cos\;f\right)\left[\begin{array}{l} \sin\;f+e\;\sin\;f\;\cos\;f+\\ \left(e+2\cos\;f+e\;\cos^{2}\;f-3\sin\;f-3e\;\sin\;f\;\cos\;f\right)\sin^{2}i\;\sin\;2u \end{array}\right]\\ & =-\frac{3{R_{e}}^{2}J_{2}}{2p^{2}}\left(1+e\;\cos\;f\right)\left[\begin{array}{l} \sin f\left(1+e\;\cos\;f\right)+\\ \sin^{2}i\;\sin\;2u\left(e+\cos\;f+\cos\;f\left(1+e\;\cos\;f\right)-3\sin\;f\left(1+e\;\cos\;f\right)\right) \end{array}\right]\\ & =-\frac{3{R_{e}}^{2}J_{2}}{2p^{2}}{\left(1+e\;\cos\;f\right)}^{2}\left[\sin\;f+\sin^{2}i\;\sin\;2u\left(\cos\;f-3\sin\;f+\frac{e+\cos\;f}{1+e\;\cos\;f}\right)\right] \end{array}$$

(A4) $$\begin{array}{ll} \frac{di}{df} & =-\frac{p^{2}}{\mu }\frac{\cos\left(f+\omega \right)}{{\left(1+e\;\cos\;f\right)}^{3}}\frac{3\mu {R_{e}}^{2}J_{2}}{2r^{4}}\sin2i\;\sin\left(f+\omega \right)\\ & =-\frac{3{R_{e}}^{2}J_{2}}{2p^{2}}\frac{p^{4}}{r^{4}}\frac{\cos\left(f+\omega \right)}{{\left(1+e\;\cos\;f\right)}^{3}}\sin2i\;\sin\left(f+\omega \right)\\ & =-\frac{3{R_{e}}^{2}J_{2}}{2p^{2}}\cos\left(f+\omega \right)\left(1+e\;\cos\;f\right)\sin2i\;\sin\left(f+\omega \right)\\ & =\frac{-3{R_{e}}^{2}J_{2}\sin2i}{2p^{2}}\left(1+e\;\cos\;f\right)\sin\;u\;\cos\;u \end{array}$$

(A5) $$\begin{array}{ll} \frac{d\Omega }{df} & =-\frac{p^{2}}{\mu }\frac{1}{\sin\;i}\frac{\sin\left(f+\omega \right)}{{\left(1+e\;\cos\;f\right)}^{3}}\frac{3\mu {R_{e}}^{2}J_{2}}{2r^{4}}\sin2i\;\sin\left(f+\omega \right)\\ & =-\frac{3{R_{e}}^{2}J_{2}}{2p^{2}}\frac{p^{4}}{r^{4}}\frac{1}{{\left(1+e\;\cos\;f\right)}^{3}}\sin\left(f+\omega \right)\frac{1}{\sin\;i}\sin2i\;\sin\left(f+\omega \right)\\ & =-\frac{3{R_{e}}^{2}J_{2}}{2p^{2}}\left(1+e\;\cos\;f\right)\sin\;u\frac{2\sin\;i\;\cos\;i}{\sin\;i}\sin\;u\\ & =\frac{-3{R_{e}}^{2}J_{2}\cos\;i}{p^{2}}\left(1+e\;\cos\;f\right)\sin^{2}\;u \end{array}$$

(A6) $$\begin{array}{ll} \frac{d\omega }{df} & =\frac{p^{2}}{\mu }\left[\begin{array}{l} \frac{\cos\;f}{e{\left(1+e\;\cos\;f\right)}^{2}}\frac{3\mu {R_{e}}^{2}J_{2}}{2r^{4}}\left(1-3\sin^{2}i\;\sin2\left(f+\omega \right)\right)\\ -\frac{\left(2+e\;\cos\;f\right)\sin\;f}{e{\left(1+e\;\cos\;f\right)}^{3}}\frac{3\mu {R_{e}}^{2}J_{2}}{2r^{4}}\sin^{2}i\;\sin2\left(f+\omega \right)\\ +\frac{1}{\tan\;i}\frac{\sin\left(f+\omega \right)}{{\left(1+e\;\cos\;f\right)}^{3}}\frac{3\mu {R_{e}}^{2}J_{2}}{2r^{4}}\sin2i\;\sin\left(f+\omega \right) \end{array}\right]\\ & =\frac{p^{2}}{\mu }\frac{3\mu {R_{e}}^{2}J_{2}}{2r^{4}}\frac{1}{{\left(1+e\;\cos\;f\right)}^{3}}\left[\begin{array}{l} \frac{\cos\;f\left(1+e\;\cos\;f\right)}{e}\left(1-3\sin^{2}i\;\sin2\left(f+\omega \right)\right)\\ -\frac{\left(2+e\;\cos\;f\right)\sin\;f}{e}\sin^{2}i\;\sin2\left(f+\omega \right)\\ +\frac{1}{\tan\;i}\sin\left(f+\omega \right)\sin2i\;\sin\left(f+\omega \right) \end{array}\right]\\ & =\frac{3{R_{e}}^{2}J_{2}}{2p^{2}}\frac{p^{4}}{r^{4}}\frac{1}{{\left(1+e\;\cos\;f\right)}^{3}}\left[\begin{array}{l} \frac{\cos\;f\left(1+e\;\cos\;f\right)}{e}\left(1-3\sin^{2}i\;\sin2\left(f+\omega \right)\right)\\ -\frac{\left(2+e\;\cos\;f\right)\sin\;f}{e}\sin^{2}i\;\sin2\left(f+\omega \right)\\ +\frac{1}{\tan\;i}\sin\left(f+\omega \right)\sin2i\;\sin\left(f+\omega \right) \end{array}\right]\\ & =\frac{3{R_{e}}^{2}J_{2}}{2p^{2}}\left(1+e\;\cos\;f\right)\left[\begin{array}{l} \frac{\cos\;f\left(1+e\;\cos\;f\right)}{e}-3\sin^{2}i\;\sin\;2u\frac{\cos\;f\left(1+e\;\cos\;f\right)}{e}\\ -\frac{\left(2+e\;\cos\;f\right)\sin\;f}{e}\sin^{2}i\;\sin\;2u\\ +\frac{\cos\;i}{\sin\;i}2\sin\;i\;\cos\;i\sin^{2}u \end{array}\right]\\ & =\frac{-3{R_{e}}^{2}J_{2}}{2p^{2}}\left(1+e\;\cos\;f\right)\cdot \left[\begin{array}{l} \frac{\left(1+e\;\cos\;f\right)\left(\sin\;f+\cos\;f\right)+\sin\;f}{e}\sin^{2}i\;\sin\;2u\\ -\frac{\cos\;f\left(1+e\;\cos\;f\right)}{e}-2\sin^{2}\;u\cos^{2}\;i \end{array}\right] \end{array}$$

where u=f+ω.

Details of the derivations of Equations (32)–(34) are as follows. The slant range can be expressed as:

(A7) $$\begin{array}{ll} R^{2} & =r^{2}+{R_{e}}^{2}-2R_{e}r\;\cos\;\alpha_{0}\\ & ={R_{e}}^{2}+r\left(r-2R_{e}\;\cos\;\alpha_{0}\right) \end{array}$$

Taking the partial differential operations for Equation (A7), we can obtain:

(A8) $$\begin{array}{ll} 2R\dot{R} & =\dot{r}\left(r-2R_{e}\;\cos\;\alpha_{0}\right)+r\dot{r}\\ & =2\dot{r}\left(r-R_{e}\;\cos\;\alpha_{0}\right). \end{array}$$

The derivatives of *r* can be calculated as:

(A9) $$\begin{array}{ll} \dot{r} & =\frac{a\left(1-e^{2}\right)e\;\sin\;f}{{\left(1+e\;\cos\;f\right)}^{2}}\dot{\alpha }\\ & =r^{2}\dot{\alpha }\frac{e\;\sin\;f}{p}\\ & =\sqrt{\frac{\mu }{p}}e\;\sin\;f, \end{array}$$

(A10) $$\begin{array}{ll} \ddot{r} & =\dot{\alpha }\sqrt{\frac{\mu }{p}}e\;\cos\;f\\ & =\frac{\sqrt{\mu p}}{r^{2}}\sqrt{\frac{\mu }{p}}e\;\cos\;f\\ & =\frac{\mu }{r^{2}}e\;\cos\;f. \end{array}$$

In the deviations, $\dot{\alpha }$ is the satellite angular velocity. We will also use the relationships $p=a\left(1-e^{2}\right)$ and $r^{2}\dot{\alpha }=\sqrt{\mu p}$ [25]; Equation (A8) can be transformed as:

(A11) $$\frac{dR}{dt}=\dot{R}=\sqrt{\frac{\mu }{p}}\cdot \frac{e\;\sin\;f}{R}\left(r-R_{e}\;\cos\;\alpha_{0}\right).$$

Similarly, we will take the second derivatives at both sides of Equation (A7), and we get:

(A12) $${\dot{R}}^{2}+R\ddot{R}=\ddot{r}\left(r-R_{e}\;\cos\;\alpha_{0}\right)+{\dot{r}}^{2}$$

Therefore, the second derivative of *R* can be expressed as:

(A13) $$\begin{array}{ll} \ddot{R} & =\frac{\left(\frac{\mu }{r^{2}}e\;\cos\;f\right)\left(r-R_{e}\;\cos\;\alpha_{0}\right)+{\left(\sqrt{\frac{\mu }{p}}e\;\sin\;f\right)}^{2}}{R}-\frac{{\dot{R}}^{2}}{R}\\ & =\frac{\frac{\mu r}{r^{2}}e\;\cos\;f-\frac{\mu R_{e}}{r^{2}}e\;\cos\;f\cos\alpha_{0}+\frac{\mu }{p}e^{2}\sin^{2}\;f}{R}-\frac{{\dot{R}}^{2}}{R}\\ & =\frac{\frac{\mu }{p}e\;\cos\;f\left(1+e\;\cos\;f\right)+\frac{\mu }{p}e^{2}\;\sin^{2}\;f-\frac{\mu R_{e}}{r^{2}}e\;\cos\;f\;\cos\;\alpha_{0}}{R}-\frac{{\dot{R}}^{2}}{R}\\ & =\frac{\frac{\mu }{p}\left(e\;\cos\;f+e^{2}\right)-\frac{\mu R_{e}}{r^{2}}e\;\cos\;f\;\cos\;\alpha_{0}}{R}-\frac{{\dot{R}}^{2}}{R} \end{array}$$

Then, we obtain:

(A14) $$\frac{d^{2}R}{dt^{2}}=\ddot{R}=\frac{\mu }{pR}\left(e^{2}+e\;\cos\;f\right)-\frac{\mu }{r^{2}R}R_{e}\;\cos\;\alpha_{0}e\;\cos\;f-\frac{{\dot{R}}^{2}}{R}$$

Similarly, we can obtain the third derivative of *R* as Equation (34). Here, we will first use the third derivative of *r*, which is:

(A15) $$\begin{array}{ll} \dddot{r} & =-\frac{2\mu \dot{r}}{r^{3}}e\;\cos\;f-\frac{\mu }{r^{2}}e\dot{\alpha }\sin\;f\\ & =-\frac{\mu e}{r^{3}}\left(2\dot{r}\cos\;f+r\dot{\alpha }\sin\;f\right)\\ & =-\frac{\mu e}{r^{3}}\left(2\sqrt{\frac{\mu }{p}}e\;\sin\;f\;\cos\;f+\frac{\sqrt{\mu p}}{r}\sin\;f\right)\\ & =-\frac{\mu e\sin\;f}{r^{3}}\sqrt{\frac{\mu }{p}}\left(3e\;\cos\;f+1\right) \end{array}$$

When considering the small variation of Δa, Equations (41)–(43) can be derived as:

(A16) $$\begin{array}{l} \Delta C_{1a}\approx \frac{dC_{1}}{da}\Delta a\\ =\mu^{1/2}e\;\sin\;f_{0}\cos\theta_{L}\frac{d{r_{0}}^{-1/2}}{da}\Delta a\\ =-\frac{1}{2}\cdot \left(\mu^{1/2}e\;\sin\;f_{0}\cos\theta_{L}\cdot {r_{0}}^{-1/2}\right)\cdot \frac{dr_{0}}{r_{0}da}\Delta a\\ =-\frac{1}{2}\cdot C_{1}\cdot \frac{\left(1-e^{2}\right)/\left(1+e\;\cos\;f_{0}\right)}{r_{0}}\cdot \Delta a\\ =-\frac{1}{2}\cdot \frac{C_{1}}{a}\cdot \Delta a \end{array}$$

(A17) $$\begin{array}{l} \Delta C_{2a}\approx \frac{dC_{2}}{da}\Delta a\\ =\frac{1}{2}\mu e\;\cos\;f_{0}\cos\theta_{L}\cdot \left(-2\right)\cdot {r_{0}}^{-2}\cdot {r_{0}}^{-1}\frac{dr_{0}}{da}\Delta a\\ =-C_{2}\cdot \frac{dr_{0}}{r_{0}da}\Delta a\\ =-\frac{C_{2}}{a}\cdot \Delta a \end{array}$$

(A18) $$\begin{array}{l} \Delta C_{3a}\approx \frac{dC_{3}}{da}\Delta a\\ =\frac{7}{12}\mu^{3/2}e\;\sin\;f_{0}\cos\theta_{L}\cdot {r_{0}}^{-7/2}\cdot \cdot {r_{0}}^{-1}\frac{dr_{0}}{da}\Delta a\\ =\frac{7}{12}\cdot \frac{C_{3}}{a}\cdot \Delta a \end{array}$$

Similarly, when considering the small variation of Δe, Equations (44)–(46) can be derived as:

(A19) $$\begin{array}{l} \Delta C_{1e}\approx \frac{dC_{1}}{de}\Delta e\\ =\mu^{1/2}\sin\;f_{0}\;\cos\theta_{L}\frac{d\left(e{r_{0}}^{-1/2}\right)}{de}\Delta e\\ =\mu^{1/2}\sin\;f_{0}\;\cos\theta_{L}\frac{\left({r_{0}}^{-1/2}de+ed{r_{0}}^{-1/2}\right)}{de}\Delta e\\ =\mu^{1/2}\sin\;f_{0}\;\cos\theta_{L}\frac{\left({r_{0}}^{-1/2}de-\frac{1}{2{r_{0}}^{3/2}}edr_{0}\right)}{de}\Delta e\\ =C_{1}\left(\frac{1}{e}-\frac{1}{2r_{0}}\cdot \frac{dr_{0}}{de}\right)\Delta e \end{array}$$

(A20) $$\begin{array}{l} \Delta C_{2e}\approx \frac{dC_{2}}{de}\Delta e\\ =\mu \frac{\cos\;f_{0}\;\cos\theta_{L}}{2}\frac{d\left(e{r_{0}}^{-2}\right)}{de}\Delta e\\ =\mu \frac{\cos\;f_{0}\;\cos\theta_{L}}{2}\frac{\left({r_{0}}^{-2}de-2e{r_{0}}^{-3}dr_{0}\right)}{de}\Delta e\\ =C_{2}\left(\frac{1}{e}-\frac{2}{r_{0}}\cdot \frac{dr_{0}}{de}\right)\Delta e \end{array}$$

(A21) $$\begin{array}{l} \Delta C_{3e}\approx \frac{dC_{3}}{de}\Delta e\\ =-\mu^{3/2}\frac{\sin\;f_{0}\;\cos\theta_{L}}{6}\frac{d\left(e{r_{0}}^{-7/2}\right)}{de}\Delta e\\ =-\mu^{3/2}\frac{\sin\;f_{0}\;\cos\theta_{L}}{6}\frac{\left({r_{0}}^{-7/2}de-\frac{7}{2{r_{0}}^{9/2}}dr_{0}\right)}{de}\Delta e\\ =C_{3}\left(\frac{1}{e}-\frac{7}{2r_{0}}\cdot \frac{dr_{0}}{de}\right)\Delta e \end{array}$$

## References

[1] Tomiyasu K., Pacelli J.L. (1983). Synthetic aperture radar imaging from an inclined geosynchronous orbit. IEEE Trans. Geosci. Remote Sens..

[2] Guarnieri A.M., Broquetas A., Recchia A., Rocca F., Ruiz-Rodon J. (2015). Advanced radar geosynchronous observation system: Argos. IEEE Geosci. Remote Sens. Lett..

[3] Hobbs S., Guarnieri A.M., Wadge G., Schulz D. Geostare initial mission design. Proceedings of the 2014 IEEE International Geoscience and Remote Sensing Symposium (IGARSS).

[4] Madsen S.N., Chen C., Edelstein W. Radar options for global earthquake monitoring. Proceedings of the IGARSS ’02. 2002 IEEE International Geoscience and Remote Sensing Symposium.

[5] Hobbs S., Mitchell C., Forte B., Holley R., Snapir B., Whittaker P. (2014). System design for geosynchronous synthetic aperture radar missions. IEEE Trans. Geosci. Remote Sens..

[6] Ruiz-Rodon J., Broquetas A., Makhoul E., Monti Guarnieri A., Rocca F. (2014). Nearly zero inclination geosynchronous sar mission analysis with long integration time for earth observation. IEEE Trans. Geosci. Remote Sens..

[7] Hu C., Long T., Zeng T., Liu F., Liu Z. (2011). The accurate focusing and resolution analysis method in geosynchronous sar. IEEE Trans. Geosci. Remote Sens..

[8] Pi X., Freeman A., Chapman B., Rosen P., Li Z. (2011). Imaging ionospheric inhomogeneities using spaceborne synthetic aperture radar. J. Geophys. Res. Space Phys..

[9] Hu C., Tian Y., Zeng T., Long T., Dong X. (2016). Adaptive Secondary Range Compression Algorithm in Geosynchronous SAR. IEEE J. Sel. Top. Appl. Earth Obs. Remote Sens..

[10] Li D., Wu M., Sun Z., He F., Dong Z. (2015). Modeling and processing of two-dimensional spatial-variant geosynchronous sar data. IEEE J. Sel. Top. Appl. Earth Obs. Remote Sens..

[11] Bruno D., Hobbs S.E. (2010). Radar imaging from geosynchronous orbit: Temporal decorrelation aspects. IEEE Trans. Geosci. Remote Sens..

[12] Rodon J.R., Broquetas A., Guarnieri A.M., Rocca F. (2013). Geosynchronous SAR focusing with atmospheric phase screen retrieval and compensation. IEEE Trans. Geosci. Remote Sens..

[13] Dong X., Hu C., Tian W., Tian Y., Long T. (2015). Design of validation experiment for analysing impacts of background ionosphere on geosynchronous sar using GPS signals. Electron. Lett..

[14] Tian Y., Hu C., Dong X., Zeng T., Long T., Lin K., Zhang X. (2015). Theoretical analysis and verification of time variation of background ionosphere on geosynchronous sar imaging. IEEE Geosci. Remote Sens. Lett..

[15] Hu C., Li X., Long T., Gao Y. GEO SAR interferometry: Theory and feasibility study. Proceedings of the 2013 IET International Radar Conference.

[16] Hu C., Li Y., Dong X., Long T. (2015). Optimal data acquisition and height retrieval in repeat-track geosynchronous sar interferometry. Remote Sens..

[17] Kou L.-L., Wang X.-Q., Xiang M.-S., Chong J.-S., Zhu M.-H. (2011). Effect of orbital errors on the geosynchronous circular synthetic aperture radar imaging and interferometric processing. J. Zhejiang Univ. Sci. C.

[18] Jiang M., Hu W., Ding C., Liu G. (2015). The effects of orbital perturbation on geosynchronous synthetic aperture radar imaging. IEEE Geosci. Remote Sens. Lett..

[19] Zhao B., Qi X., Song H., Wang R., Mo Y., Zheng S. (2014). An accurate range model based on the fourth-order doppler parameters for geosynchronous sar. IEEE Geosci. Remote Sens. Lett..

[20] George W., Collins I. (2004). The Foundations of Celestial Mechanics.

[21] Delgado M.R. (2008). Modeling the Space Environment.

[22] Montenbruck O., Gill E. (2000). Satellite Orbits: Models, Methods and Applications.

[23] Pavlis N., Holmes S., Kenyon S., Factor J. (2012). The development and evaluation of the Earth Gravitational Model 2008 (EGM2008). J. Geophys. Res..

[24] Hu C., Long T., Liu Z., Zeng T., Tian Y. (2014). An improved frequency domain focusing method in geosynchronous sar. IEEE Trans. Geosci. Remote Sens..

[25] Curlander J.C., Macdonough R.N. (1991). Synthetic Aperture Radar: Systems and Signal Processing.

